# The Impact of Plant Enemies Shows a Phylogenetic Signal

**DOI:** 10.1371/journal.pone.0123758

**Published:** 2015-04-20

**Authors:** Gregory S. Gilbert, Heather M. Briggs, Roger Magarey

**Affiliations:** 1 Environmental Studies Department, University of California Santa Cruz, Santa Cruz, California, United States of America; 2 Smithsonian Tropical Research Institute, Balboa, Ancón, Republic of Panama; 3 Center for Integrated Pest Management, North Carolina State University, Raleigh, North Carolina, United States of America; Julius Kuehn-Institute (JKI), GERMANY

## Abstract

The host ranges of plant pathogens and herbivores are phylogenetically constrained, so that closely related plant species are more likely to share pests and pathogens. Here we conducted a reanalysis of data from published experimental studies to test whether the severity of host-enemy interactions follows a similar phylogenetic signal. The impact of herbivores and pathogens on their host plants declined steadily with phylogenetic distance from the most severely affected focal hosts. The steepness of this phylogenetic signal was similar to that previously measured for binary-response host ranges. Enemy behavior and development showed similar, but weaker phylogenetic signal, with oviposition and growth rates declining with evolutionary distance from optimal hosts. Phylogenetic distance is an informative surrogate for estimating the likely impacts of a pest or pathogen on potential plant hosts, and may be particularly useful in early assessing risk from emergent plant pests, where critical decisions must be made with incomplete host records.

## Introduction

The outcome of interactions between plants and their enemies depends on traits of both organisms [1–3]. Plant traits commonly show a phylogenetic signal, where close relatives are more likely to have similar traits [4–6]. Many traits important in plant-enemy interactions show such phylogenetic signals [7–13], although there are exceptions [12, 14–16]. When a plant pathogen or pest has the necessary traits to overcome or avoid particular defenses and successfully attack a particular plant species, it may be better able to attack closely related plant species that share the same, phylogenetically conserved, defense traits. Indeed, the host ranges of most plant pests and pathogens show a clear phylogenetic signal, where the probability that two plants species will share a particular pest declines steadily with phylogenetic distance between them [17–20].

Neither the potential nor realized host ranges of pests and pathogens are ever completely known, yet knowledge of the host range of an enemy is fundamental to understanding its spread, evolution, and impacts in natural and managed ecosystems. For instance, phytosanitary risk analysis of ecological and economic threats from novel or emergent pests are by necessity conducted before adequate empirical data on host ranges are available [21], and risk assessment of potential biological control agents requires careful, time-demanding empirical testing of local non-target hosts [22]. In such cases, phylogenetic models based on evolutionary distances from known hosts of a pest to local plant species of concern can point to which species are most likely to be susceptible hosts, serving to guide priorities for examination [23]. Similarly, forest restoration, agroforestry, and other multi-species managed systems can use models based on the phylogenetic signal in host range to help select combinations of species to grow together that are less likely to share pests or pathogens [24]. Phylogenetic relationships shape the breadth and structure of host ranges of pathogens and parasites in natural communities, which in turn have important impacts on host community diversity, structure, and dynamics [25–28]

Existing models of the phylogenetic signal in host range have relied mostly on binary measures of the host-enemy interaction (susceptible or resistant) [18, 19, 29, 30]. These empirically derived models provide a quantitative estimate of the probability that two host species will share a particular enemy, given the phylogenetic distance between the hosts. It is still unclear whether the strength of pest impact will follow a similar phylogenetic signal. In fact, binary models might overestimate the risk to plant species at moderate phylogenetic distances. We might expect severity of impact to drop off more quickly with phylogenetic distance (a steeper phylogenetic signal) if small deviations from a particular favorable constellation of traits deprive a pest from being able to thrive on and damage a host. Such trait aggregates may only be found on very closely related species, and small deviations may lead to reduced severity before it leads to qualitative (binary) resistance. In such a case, the set of highly susceptible hosts would be narrower and more closely related than is the full set of susceptible hosts. It is therefore important to measure the strength and steepness of phylogenetic signal in the impact of plant-enemy interactions.

There are two kinds of quantitative impacts that may show phylogenetic signal and that are important in evaluating the potential risk posed by a pest or pathogen. The first is the severity of impact of the enemy on the plant, such as amount of biomass consumed, leaf area lost to lesions, or the proportion of seeds attacked. The second is the impact on the enemy itself. This may be further subdivided into measures of performance effects (growth rate, reproduction, survival) and measures of preference (which hosts are selected for oviposition). We used published literature with quantitative measures of these impacts to evaluate the strength and slope of phylogenetic signal in the severity of impacts of plant pathogens and pests for five categories of effects.

We extracted data from published studies on the strength of impacts of plant pathogens and herbivores across a diversity of host plants and reanalyzed them within an explicitly phylogenetic framework. We found quantitative data for five categories: (1) impact of pathogen on the host plant, (2) impact of herbivore on the host plant, (3) impact of host on pathogen performance, (4) impact of host on herbivore performance, and (5) impact of host on herbivore preference behavior. For each study, we first measured the phylogenetic signal in the relative impact of particular enemies across a diversity of plant species. We then conducted a meta-analysis of the resulting regression models to examine the overall phylogenetic signal for each of five categories of severity of impact of plant pathogens and pests.

## Methods

### Data sources

We sought published literature with quantitative measures of plant-enemy interactions where the effects were measured on a phylogenetic diversity of host plant species within the same study. We searched Web of Science, followed references cited in papers we found, and asked colleagues for recommended data sets. The initial search included the search: plant AND (herbiv* OR parasit* OR pathogen*) AND ("host range" or "host plant shift" or "host preference”), which resulted in 1494 records in Dec 2012. Of these, the vast majority clearly lacked appropriate data based on review of keywords and titles. 127 publications (plus three others recommended by colleagues through April 2013) were more carefully screened for eligible data. To qualify, a publication needed to have (1) a quantitative measure of impact on the host or on the enemy (pathogen or herbivore), (2) a minimum of five plant species belonging to multiple Angiosperm plant families, and (3) more than one plant species had to have a non-zero response to the enemy. All but 15 of the 130 published studies we found on enemy impact across plant species were not suitable for our analysis either because the measures of impact were not quantitative or because the range of hosts tests was very narrow (e.g., several cultivars within a crop species, or several species within a genus). Suitable publications included experimental tests in lab and field setting from around the world, including a great diversity of agronomic and wild host species. Studies included enemy-plant pairs with long histories of association as well as novel interactions. This diversity provides a broad sampling of kinds of enemy-host interactions from diverse settings. When multiple response variables were presented within a publication, each response was treated as a separate study unit. In total we found 15 suitable publications (4 on pathogens and 11 on herbivores) that collectively provided 48 study units and 989 observations on 228 Angiosperm plant and 26 enemy taxa (S1 Table). Host diversity within a study unit ranged from 5 to 65 species. Seven study units evaluated the impact on enemy development (e.g., spore production, larval growth), 5 on enemy behavior (e.g., oviposition), and 29 on the host plant (e.g., lesion size, biomass consumed) (S1 Table).

### Phylogenetic distances

For each study unit we calculated the phylogenetic distance (as millions of years (Ma) of independent evolution) from the most impacted host (the focal host) to every other host, following the approach used in Gilbert and Webb [19]. We used the Phylomatic commands in Phylocom v 4.2 (http://phylodiversity.net/phylocom/) to calculate expected pairwise phylogenetic distances among all 228 plant taxa. We base our phylogenetic tree on a hand-constructed supertree R2G2_20140601 which includes a topology of phylogenetic relationships of all vascular plant families, and within-family substructure for all major families of plants for which accepted topologies were available [31]. The Angiosperm phylogeny follows the APGIII system [32]. The tree was dated based on an expanded and updated set of the minimum node ages given in Wikstrom et al. [33]. We used the bladj function in Phylomatic and to provide estimates of phylogenetic distance between pairs of host taxa. Although there are more refined methods available to estimate phylogenetic distances based on molecular phylogenies, we chose to use the Wikstrom dating approach to provide continuity and comparability to previous studies [18, 19] and because it allows inclusion any Angiosperm plant taxa, even when molecular phylogenetic data are not available. The dated tree used for analyses is presented in S1 Newick Tree.

### Within-study unit regressions

For each study unit we identified a focal plant species—that for which the measured response was strongest. We chose the most strongly affected host as the focal host rather than the most common host because which hosts are most commonly attacked is likely to vary temporally and geographically, and because the phylogenetically constrained traits that are most likely to affect severity of plant-enemy interactions are more likely to be density independent [i.e., chemical and structural). When there were multiple possible focal hosts with the same maximum value, we randomly selected one as the focal host. We calculated the phylogenetic distance from that focal host to each other host in the study unit as described above. We calculated the relative effect on each host by dividing the response by that of the focal host, so that the impact was always positive and ranged up to one. We then calculated a linear regression with the model RelativeEffect = *b*_0_ + *b*_1_ * log_10_phylogenetic distance +1), where the phylogenetic distance is in millions of years (Ma) of independent evolution (twice the time to most recent common ancestor). We used the logarithmic transformation of the phylogenetic distance because the strong majority of studies were better fit with a log transformation than a linear model, and a linear model was almost never a better fit. In addition, this enabled direct comparison to published analysis of phylogenetic effects on host range, where the log transformation was clearly best [18]. We recorded the intercept, slope, coefficient standard errors, sample size, *t*-value and *p*-value for the test that the slope was different from zero, and the 95% confidence intervals for the slopes.

### Meta-analysis

We used tools from meta-analysis to then combine the results of our re-analysis of published data from each of the studies. Weighted means of the regression coefficients were calculated for each of five categories of study unit: impact of pathogen on host plant (n = 15); impact of herbivore on host plant (n = 14); effect of host on pathogen performance (n = 1); effect on host plant on herbivore performance (development) (n = 6); and the effect of host plant on herbivore behavior (n = 5). We used a weighted least squares approach to find the combined intercept (*b*_*•*0_) and combined slope (*b*_*•*1_), and following Becker and Wu [34]: *b*_•1_ = Σ(w_i1_b_i1_) / Σw_i1,_ where *b*_*i*1_ is the slope and *w*_*i*1_ is the slope weight for study *i*. The slope weight is the reciprocal of the slope variance *w*_*i*1_ = 1/Var(*b*_*i*1_). The corresponding formulas apply for the weighted mean intercept *b*_*•*0_. The variance of the slope is then Var(*b*_•1_) = 1/ Σ*w*_*i*1_.

We compare the results of these analyses to the phylogenetic signal in host range of a broad selection of plant pests already reported using binary (host/non-host) data for 95 fungal pathogens and 637 insect pests [18]. To do so, however, we first replicated the logistic regression analyses of host ranges of polyphagous pests in the original USDA Global Pest and Pathogen Database data but using the R2G2_20140601 phylogenetic tree and phylogenetic distances from the current study, so that the regression coefficients would be directly comparable. These re-analyses are presented in detail elsewhere [31].

All analyses were conducted using R version 3.0.2 (http://www.r-project.org/).

## Results

The severity of impact of plant-enemy interactions declined with increasing phylogenetic distance to host plants from highly impacted, focal host species. The slopes of regressions of all 48 study units were negative, and 35 of 48 (73%) study units were statistically significant (P≤0.05), indicating a widespread phylogenetic signal in enemy impact (S2 Table). There was a strong phylogenetic signal in relative impact for each of the five categories of studies included: plant disease 12/15 study units (80%), plant herbivory 9/12 (75%), pathogen development 1/1 (100%), herbivore development 5/9 (56%), and herbivore behavior 8/11 (73%). Graphical representations of each study, with overlaid regression lines, are presented in S1 Fig

**Fig 1:**
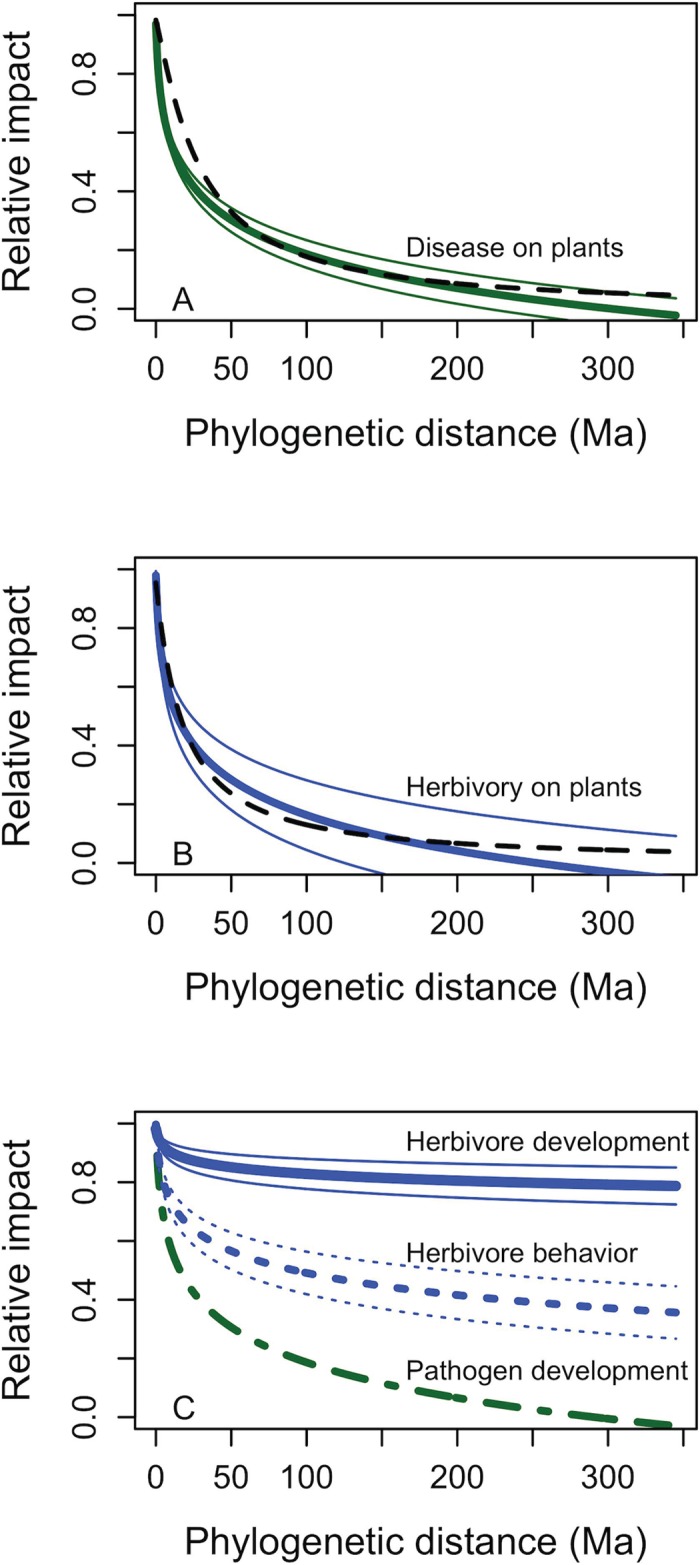
Observed phylogenetic signal in severity of impact of plant-enemy interactions. Regressions are based on weighted mean regression coefficients of relative impact on the log of the phylogenetic distance between hosts in meta-analysis of published studies. Regression coefficients are given in Table 1. Thick lines are based on mean intercept and slope, and the thin lines are 95% confidence intervals. For comparison in panels A and B, the dashed black lines show the expected probability that two hosts at that phylogenetic distance would share a particular enemy based on re-analysis of data in Gilbert et al. [18], using the present phylogenetic tree. In panel C, Pathogen development has no confidence intervals because it was derived from only one study unit (Table 1, S1 Fig, S1 Table).

The meta-analysis showed strong and consistent phylogenetic signals in the severity of impact in plant-enemy interactions (Fig 1, Table 1). For the four categories for which there were multiple studies, the slopes were all significantly negative (95% confidence intervals did not overlap zero; Table 1). For impact on pathogen development (N = 1, study unit 32h), the original slope was significantly negative (S2 Table). The strengths of phylogenetic signal for herbivore and pathogen damage on plants were very similar, with broadly overlapping 95% confidence intervals. The phylogenetic signals for herbivore development and herbivore behavior were statistically significant, but notably less strong than the impacts on the plant hosts (Table 1, Fig 1).

**Table 1:** Weighted means of intercept (*b*_•0_) and slope (*b*_•1_) from the study-unit regressions described in S2 Table, for each of five types of interaction type and measured impact.

**Interaction**	**Impacted**	**N**	**Intercept** ^**a**^	**Slope** ^**a**^	**SE intercept**	**SE slope**	**CI025** ^**c**^	**CI975**
disease	plant	15	0.9694	-0.3906	0.0231	0.0104	-0.4110	-0.3701
herbivory	plant	12	0.9792	-0.4072	0.0498	0.0265	-0.4593	-0.3552
disease	enemy development	1	0.9965	-0.4042	NA^b^	NA	NA	NA
herbivory	enemy development	9	0.9818	-0.0766	0.0201	0.0113	-0.0988	-0.0545
herbivory	enemy behavior	11	0.9961	-0.2520	0.0297	0.0162	-0.2836	-0.2203

^a^Coefficients are for the model *RI* = *b*_•0_ + *b*_•1_log_10_(*PD* + 1), where *PD* is the phylogenetic distance in Ma from the most strongly affected host.

^b^Standard errors for the weighted means are not available for disease—enemy development category because there was only one study unit (N), but were significant in the original study.

^c^The 95% confidence intervals are presented for the weighted mean slopes.

The shape and steepness of the phylogenetic signal in disease and herbivory severity are remarkably similar to those found in a much more extensive analysis of binary (host/non-host) host range data for fungal pathogens and insect pests (reanalysis of data in [18] using the current phylogenetic tree) (Fig 1). This suggests a strong similarity in the phylogenetic signal in absolute host range and in the impact of enemies on their host plants.

## Discussion

The impact of pathogens and pests on different plant species shows a strong phylogenetic signal; the relative amount of damage done by a natural enemy on plant species declines predictably with increasing evolutionary distance from highly susceptible hosts. The shape and steepness of this phylogenetic signal in damage severity is similar to that already recognized for binary measures of host range. In addition, we found a significant phylogenetic signal in performance and behavior of the enemies themselves.

Previous analysis of phylogenetic signal in host range of pathogens and pests have focused on whether or not a plant species was susceptible or resistant to the enemy, without regard to the severity of impact. This is primarily because of the much greater availability of qualitative response data on susceptibility/resistance. Models based on phylogenetic regression of these binary-response data should be useful for initial analysis of the risk from novel pests and pathogens to plant species of interest, when empirical data are not yet available [3, 18]. However, the important question remained whether such models would overestimate the potential risks to plants at moderate phylogenetic distances from known hosts—the case if enemies are able to attack a broader range of host than those on which they thrive. The similarity in the phylogenetic signal of quantitative and qualitative measures of impact on hosts (Fig 1), suggest that the risk estimates based on (the more extensively validated) models of known host ranges should provide a useful estimate of expected severity of impact on particular hosts.

The quantitative impact analysis here showed a phylogenetic signal very similar to that found in the broad analysis of host range of plant pests and pathogens from the USDA database [18] (Fig 1). Nevertheless, evolutionary history and life-history traits of the enemies can have an important influence on the strength of phylogenetic signal in particular cases. For instance, a cross-inoculation study [19] focused on foliar fungal pathogens of co-occurring plants showed a shallower slope that that shown here or in Gilbert et al. [18]. Our results, drawn from a diversity of different kinds of enemies and sets of hosts, represent an overall pattern that could be considered a null model for expected behavior, to which specific systems can then be compared.

Host-specific pests and pathogens that create negative density-dependent responses in their hosts have long been thought to be important drivers in maintenance of plant species diversity [35–38]. Our results here suggest that because most plant enemies are polyphagous but that their impacts decline with phylogenetic distance among hosts, that pests and pathogens may be important in maintaining diversity in natural systems at phylogenetic levels above species. This points to the importance of considering the full range of local hosts in studies of density-dependence in plant-enemy interactions [31].

Our analysis is limited by a paucity of appropriate available data in two primary ways. When the phylogenetic range of hosts within a study is narrow, we may underestimate the steepness of the slope of phylogenetic signal because the observed range of response may be truncated from the true range of reactions (i.e., phylogenetically distant plant species are less likely to be compatible with the enemy). Many of the studies we found that had quantitative measures of the impacts of enemies on multiple species of plants were limited to a very narrow phylogenetic range of hosts—often multiple cultivars within a species or a few closely related species. Some other studies with broader phylogenetic range included very few species. Either case limits the robust use of phylogenetic regression analysis, and such studies were excluded from analysis. Even among some of the studies with broader ranges of hosts, the phylogenetic distances were often restricted to a relatively short range (e.g., S1 Table, studies 4,9,12).

Second, the designation of the most severely impacted species as the reference to calculate phylogenetic distances means that all the phylogenetic distances are particular to the context of the range of tested species. Other, untested species may be much more severely impacted; including them in the regression would rescale the dependent axis and restructure the phylogenetic distances, and as above, would tend to underestimate the steepness of phylogenetic signal in severity. Failure to include highly compatible plant-enemy pairs (i.e., where the enemy can cause the most damage) could explain why some of the individual studies showed no significant phylogenetic signal (S2 Table). Both of these limitations in the available data should make it less likely for us to detect strong phylogenetic signal in the data, so we regard the measured strengths of phylogenetic signal reported here as conservative.

Our analytical approach includes two potential biases. First, by setting the most impacted host as focal species with a phylogenetic distance to itself of zero and a relative impact of one, then all other points will be ≤1, forcing a negative slope for the regression. However, by generating random data of the same sample sizes and phylogenetic distance ranges as found in the individual studies, we found that only 13.5% of 80,000 runs generated slopes significantly different from zero, compared to 73% of the cases presented in Supporting Information Table 1. This suggests that any bias of slope from this approach is minimal. Second, because we defined the relative impact of focal species as 1.0, it could be argued that we should fix the intercept at 1.0, and only estimate the slope. However, there are two reasons that it is more realistic and conservative to instead estimate the intercept. First, variation within species means that the value for the focal species is an estimate, rather than a true, fixed value. Second, by forcing the regression through (0,1) it would necessarily produce a more negative estimate of slope. This would have the effect of potentially overestimating expected severity for species very closely related to the focal species, but with little effect on estimates of more distantly related species. In addition, it is worth noting that in practice, estimating the intercept makes little difference: estimated mean intercepts ranged from 0.9609 to 9.997 (Table 1).

The agronomic or ecological impact of an emergent pest or pathogen depends both on the direct impact of the enemy on the affected host plant and on performance measures of the enemy (i.e., growth and reproduction) on those hosts. Such performance measures are often critical determinants of persistence and spread of enemies [39–42]. Our results show a strong, significant phylogenetic signal in enemy performance. This would be expected if the traits that govern interactions between plants and their enemies were phylogenetically constrained. Many of the traits that govern qualitative resistance/susceptibility may be the same that determine the severity of impact, although other traits may have more pronounced effects on growth and reproduction (e.g., particular nutritional content of hosts) or host selection (e.g., production of attractive volatiles) than on the amount of damage an enemy can cause on a host plant. Antonovics *et al*. [1] explored different evolutionary scenarios for non-host resistance, with particular attention to when resistance is an evolutionary response of the plant compared to when host range results from evolutionary change in the pathogen that affects host choice. Overall there is stronger evidence that non-host resistance is the product of evolutionary changes in the pests, but because the pests are responding to host traits, there remains a phylogenetic signal in host range. This is congruent with our present findings.

Phylogenetic signal is also common for animal-parasite interactions [43–46]. Interestingly, Desneux et al. [47] found much stronger phylogenetic signal in preference traits (those related to host selection) than performance traits (hatching and survival) for a wasp parasitoid of aphid species. Similarly, our analysis suggested much stronger phylogenetic signal (steeper decline with phylogenetic distance) in herbivore oviposition behavior (host preference) than in herbivore development (Fig 1). Our estimates for herbivore development, however, come from only five measures on three herbivore species, and for one of those species, the phylogenetic range of plants was rather limited. In addition, we do not have measures of both behavior and development for the same pests on the same range of host plants, limiting the strength of any comparisons. Nevertheless, this suggest that either host preferences may be too narrow, so that the pests miss opportunities for successful larval development, or that the developmental tests are insensitive to selective differences among hosts for larval development. In either case, this is a potentially interesting pattern that deserves direct experimental testing.

Questions of enemy specificity have often been framed in terms of hierarchical categories: Is a pathogen species-specific? Is host specificity of herbivores expected at the genus or family level? Unfortunately, the range of phylogenetic distances within named genera or families varies tremendously depending on the genus and family; there is as much phylogenetic range within some genera as can be found between different families. As such, direct translation from our phylogenetic distance approach to such arbitrary taxonomic categories should be viewed with caution. However, as a general guide, the results here suggest that the impact of herbivores and pathogens on plants within the same genus should be 30–50% that of the focal (most impacted) host; plants in the same family, but outside the genus should suffer 10–30% of the impact on the focal host.

Importantly, an estimate of phylogenetic distances is now easily calculated for any set of plant species based on published phylogenetic trees. Phylogenetic distance serves as a surrogate for similarity of some plant traits that are important in plant-enemy interactions. This points to phylogenetic regression models as useful tools for estimating the likely impact of a pest or pathogen on particular host species when empirical data are incomplete. Such a tool should be broadly useful for studies in plant ecology, conservation biology, biological control, agronomy, and phytosanitary risk analysis.

## Supporting Information

S1 Fig Phylogenetic signal for each of 48 study units used in this study. Regression lines reflect best fit line *RI* = *b*_•0_ + *b*_•1_log_10_(*PD* + 1), where RI is the relative impact on the host or enemy compared to the most severely affected species in the study, and PD is the phylogenetic distance from that host to the most severely affected species, in units of millions of years of independent evolution. See S1 Table for sources and S2 Table for coefficients and statistics. figures are grouped by Type of Interaction and impacted partner: S1A. Herbivore Impacts on Host Plants, S1B. Pathogen Impacts on Host Plants, S1C. Plant Impacts on Herbivore Behavior, and S1D. Plant Effects on Herbivore or Pathogen Development.
(PDF)

S1 Newick Tree Phylogenetic tree used to estimate phylogenetic distances between host plants. The tree is based on a supertree of all vascular genera (R2G2_20140601 [31]). Angiosperm topology is based on APGIII classification [32], with major nodes dated using an updated list of Wikstrom et al. [33] minimum node ages. Remaining nodes in the tree were given interpolated ages using the BLADJ function of Phylocom, and the overall structure and dating from this tree was then used as the basis for the tree of 228 plant taxa presented here.
(DOCX)

S1 Table Description of publications and study units from which data were used for phylogenetic analysis and meta-analysis. (PDF)

S2 Table Regression outputs from linear regression for each of the study units described in S1 Table. Regressions take the form of *RI* = *b*_•0_ + *b*_•1_log_10_(*PD* + 1). Phylogenetic distance (PD) is the estimated time of independent evolution from the most affected species in the study unit (in Ma, millions of years); b_0_ is the slope and b_1_ is the intercept. P-values in bold indicate slopes significantly different from zero (alpha = 0.05). Confidence intervals (95%) are given for the slope of each model. The type of interaction and which partner was impacted are used for meta-analysis grouping in Table 1 and Fig 1 in the main text.
(PDF)
