# Supplementary material for: The Impact of Plant Enemies Shows a Phylogenetic Signal
Source: PLoS One. 2015 Apr 20;10(4):e0123758. doi: 10.1371/journal.pone.0123758 (PMC4404352; doi:10.1371/journal.pone.0123758)
Supplement: S1 Fig — Regression lines reflect best fit line RI = b •0 + b •1log10(PD + 1), where RI is the relative impact on the host or enemy compared to the most severely affected species in the study, and PD is the phylogenetic distance from that host to the most severely affected species, in units of millions of years of independent evolution. See S1 Table for sources and S2 Table for coefficients and statistics. figures are grouped by Type of Interaction and impacted partner: S1A. Herbivore Impacts on Host Plants, S1B. Pathogen Impacts on Host Plants, S1C. Plant Impacts on Herbivore Behavior, and S1D. Plant Effects on Herbivore or Pathogen Development. (PDF) [file pone.0123758.s001.pdf]

Figure S1. Phylogenetic signal for each of 48 study units used in this study. Regression lines reflect best fit line $RI = b_{\bullet 0} + b_{\bullet 1}\log_{10}(PD + 1)$, where RI is the relative impact on the host or enemy compared to the most severely affected species in the study, and PD is the phylogenetic distance from that host to the most severely affected species, in units of millions of years of independent evolution. See Table S1 for sources and Table S2 for coefficients and statistics. Figures are grouped by Type of Interaction and impacted partner: S1A. Herbivore Impacts on Host Plants, S1B. Pathogen Impacts on Host Plants, S1C. Plant Impacts on Herbivore Behavior, and S1D. Plant Effects on Herbivore or Pathogen Development.

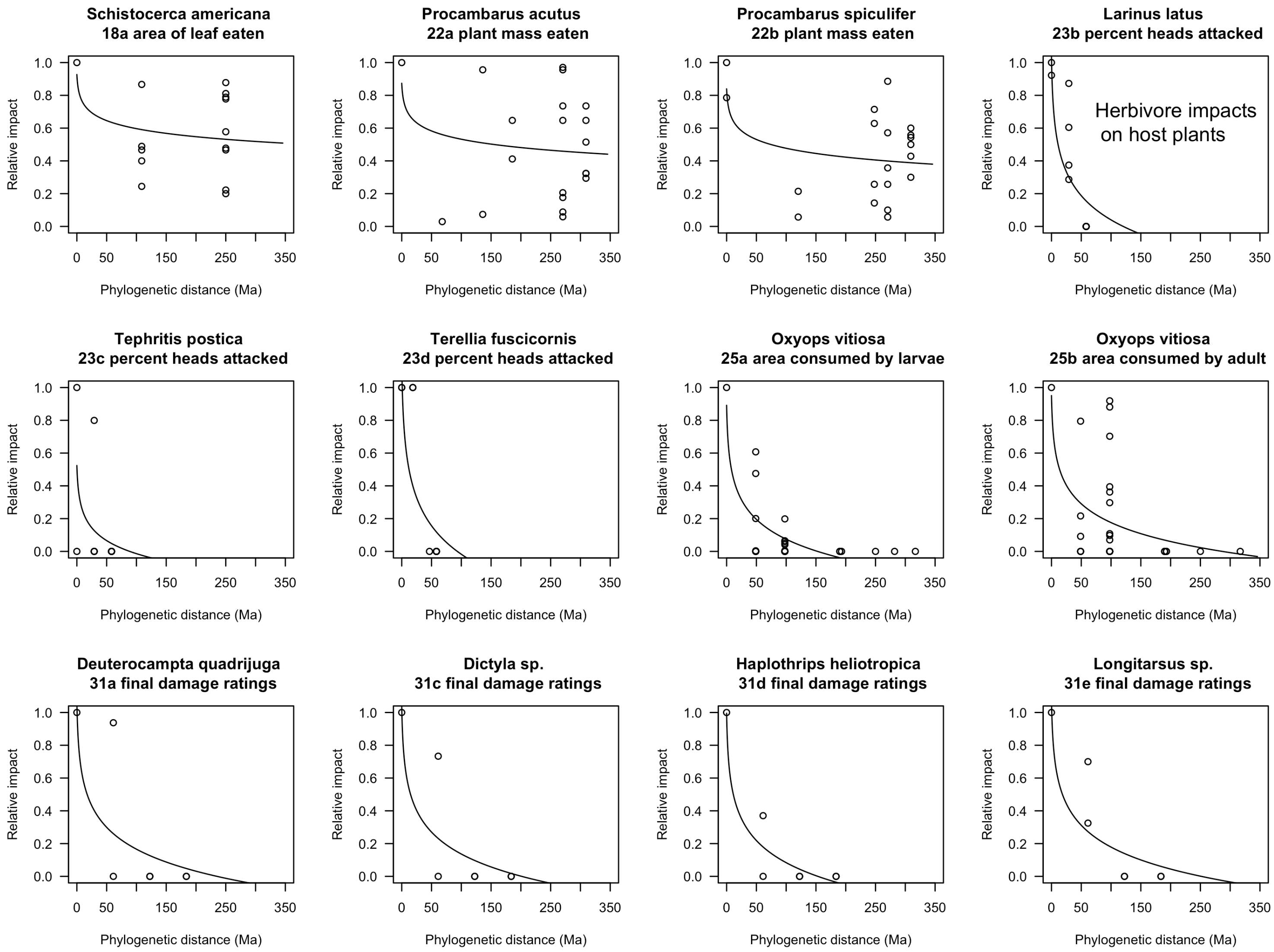

Figure 1A. Herbivore Impacts on Host Plants

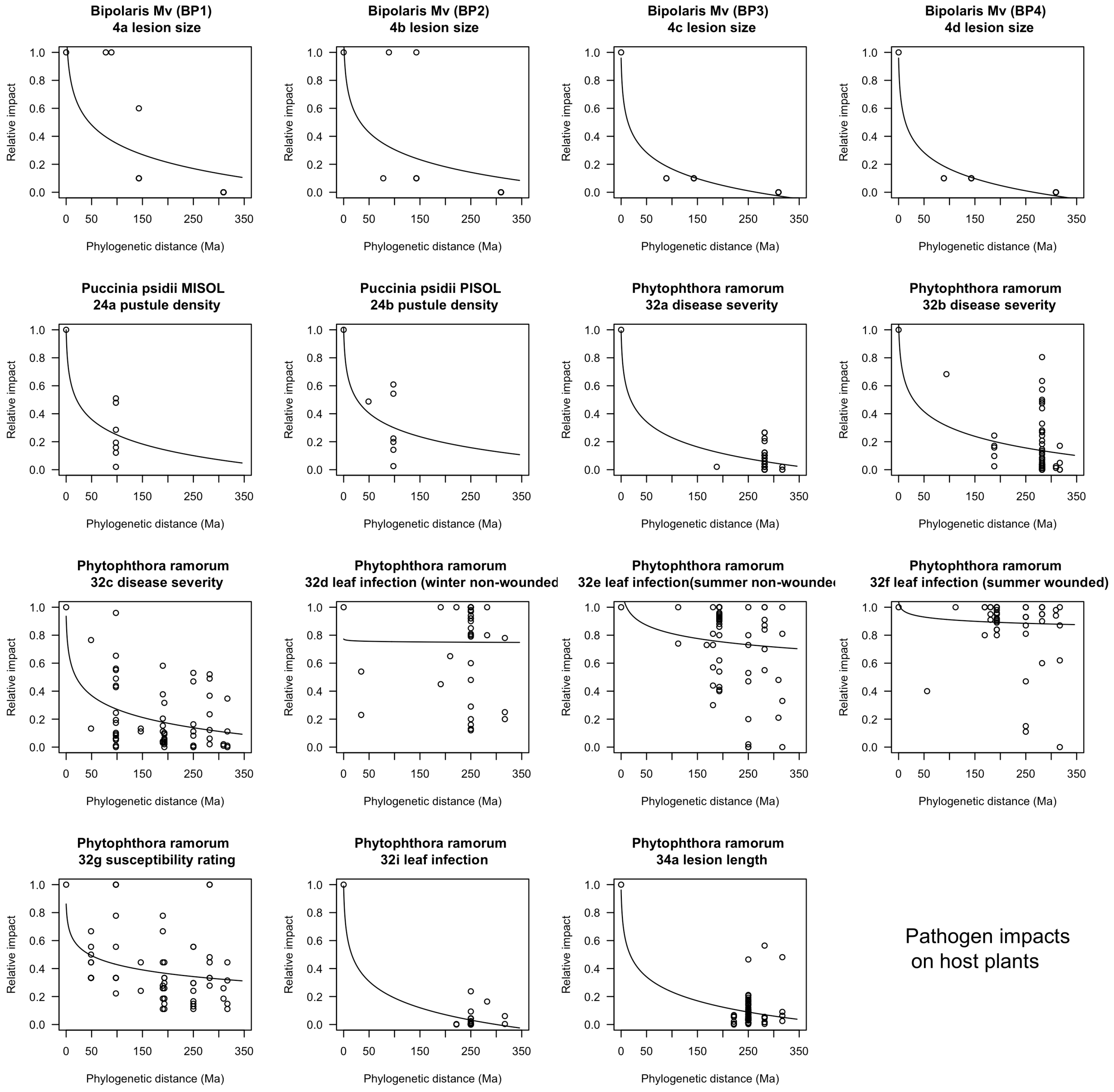

Figure 1B. Pathogen Impacts on Host Plants

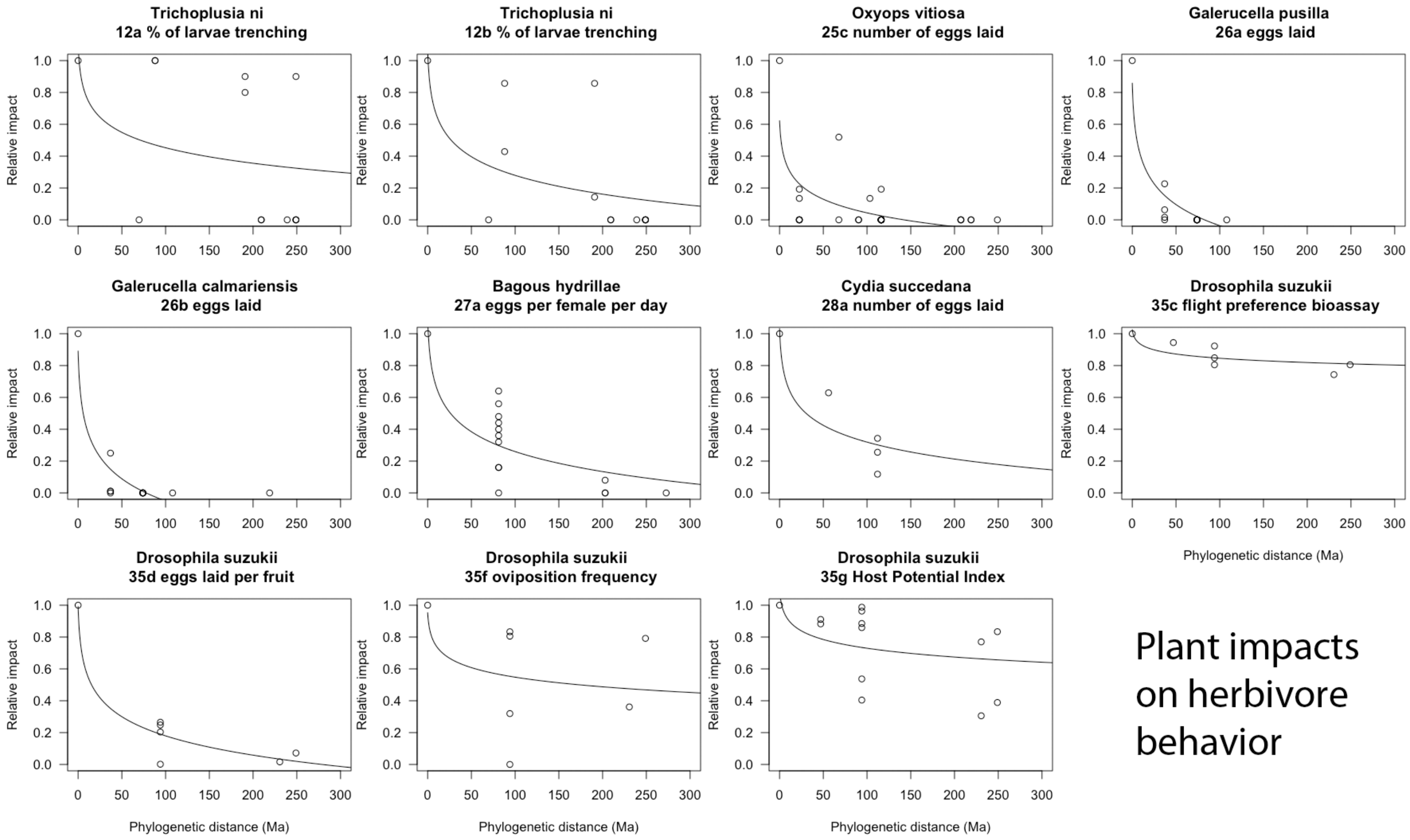

Figure S1C. Plant Impacts on Herbivore Behavior

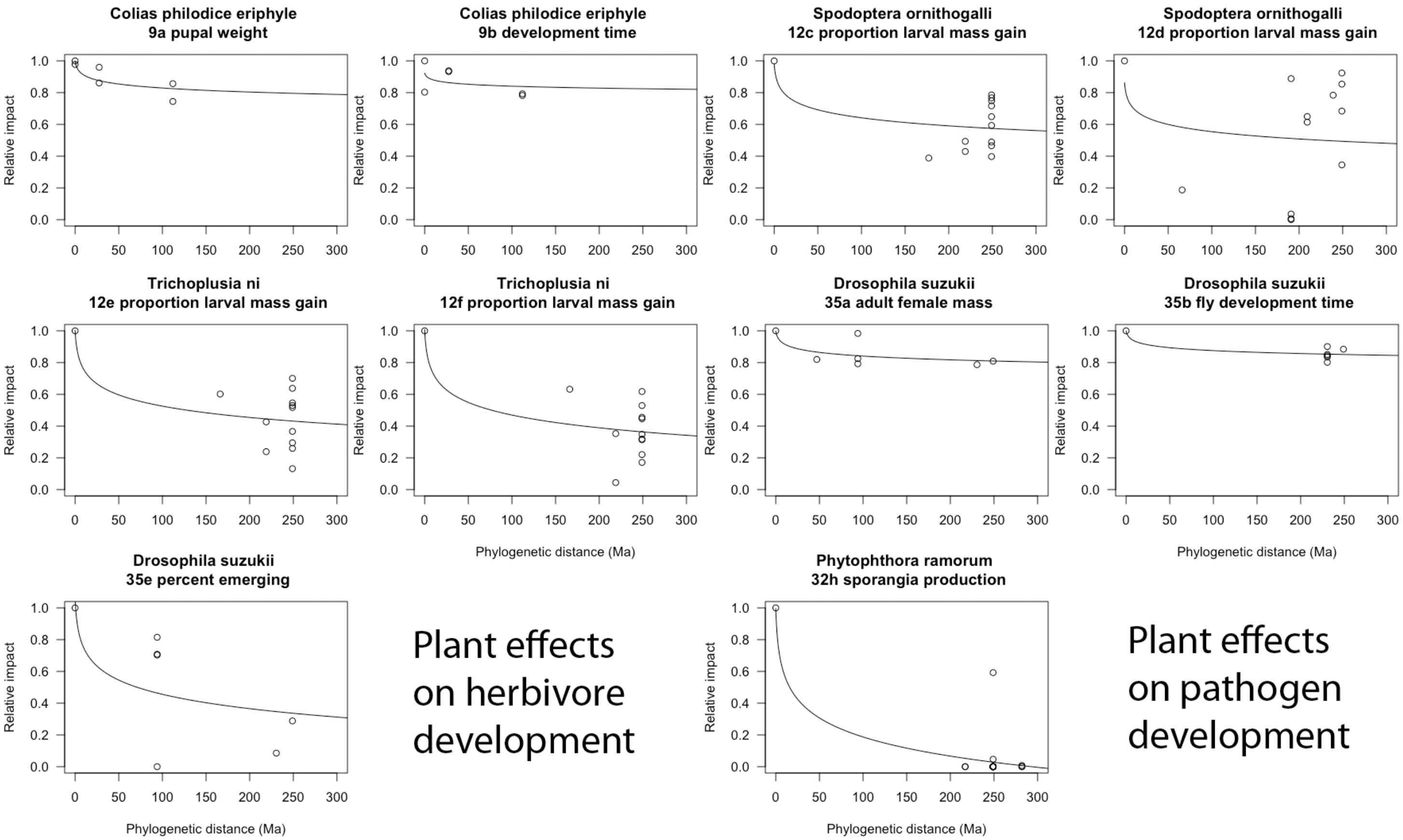

Figure S1D. Plant Effects on Herbivore or Pathogen Development
