# Supplementary material for: The Impact of Plant Enemies Shows a Phylogenetic Signal
Source: PLoS One. 2015 Apr 20;10(4):e0123758. doi: 10.1371/journal.pone.0123758 (PMC4404352; doi:10.1371/journal.pone.0123758)
Supplement: S1 Newick Tree — The tree is based on a supertree of all vascular genera (R2G2_20140601 [31]). Angiosperm topology is based on APGIII classification [32], with major nodes dated using an updated list of Wikstrom et al. [33] minimum node ages. Remaining nodes in the tree were given interpolated ages using the BLADJ function of Phylocom, and the overall structure and dating from this tree was then used as the basis for the tree of 228 plant taxa presented here. (DOCX) [file pone.0123758.s002.docx]

**Newick Tree S1**. **Phylogenetic tree used to estimate phylogenetic distances between host plants.** The tree is based on a supertree of all vascular genera (R2G2_20140601, Parker et al. *in review).* Angiosperm topology is based on APGIII classification (Bremer *et al.* 2009), with major nodes dated using an updated list of Wikstrom et al. (2001) minimum node ages. Remaining nodes in the tree were given interpolated ages using the BLADJ function of Phylocom, and the overall structure and dating from this tree was then used as the basis for the tree of 228 plant taxa presented here.

(((((((((((((acacia_harpophylla:28.000000,acacia_melanoxylon:28.000000,acacia_dealbata:28.000000,acacia_oncinocarpa:28.000000,acacia_mangium:28.000000)acacia:28.000000,(((clianthus_maximus:15.679993,((lathyrus_sp.:5.226685,vicia_sp.:5.226685)fabeae:5.226654,medicago_sativa:10.453339)n00442:5.226654)irlc:9.706665,(glycine_max:11.075562,(hardenbergia_violaceae:5.537781,hardenbergia_violacea:5.537781)hardenbergia:5.537781,phaseolus_vulgaris:11.075562)n00446:14.311096)n00450:8.213348,((sophora_tetraptera:9.199997,sophora_prostrata:9.199997)sophora:9.199997,ulex_europaeus:18.399994)n00455:15.200012)mesopapilionoideaeclade:22.399994)fabaceae:28.649994,(((citrullus_lanatus:36.331253,cucumis_sativus:36.331253)cucurbitaceae:36.331253,(nothofagus_cunninghamii:36.331253,nothofagus_moorei:36.331253)nothofagus:36.331253)n00398:7.662506,((fragaria_ananassa:50.666668,(prunus_avium:25.333334,prunus_persica:25.333334)prunus:25.333334,(rubus_fruticosus:25.333334,rubus_idaeus:25.333334)rubus:25.333334)rosaceae:25.333334,pomaderris_apetala:76.000000)rosales:4.325012)n00434:4.324982)n00463:5.649994,(((bauera_rubioides:28.033335,bauera_rubiodes:28.033335)bauera:28.033335,ceratopetalum_apetalum:56.066669,eucryphia_lucida:56.066669)cunoniaceae:28.033335,viola_hederaceae:84.100006)n00482:6.199982)n00484:6.200012,(((acmena_smithii:48.888874,agonis_flexuosa:48.888874,(callistemon_viminalis:24.444437,callistemon_pachyphyllus:24.444437,callistemon_salignus:24.444437)callistemon:24.444437,(corymbia_maculata:24.444437,corymbia_ficifolia:24.444437)corymbia:24.444437,(eucalyptus_haemastoma:24.444437,eucalyptus_cneorifolia:24.444437,eucalyptus_diversicolor:24.444437,eucalyptus_denticulata:24.444437,eucalyptus_pauciflora:24.444437,eucalyptus_saligna:24.444437,eucalyptus_viminalis:24.444437,eucalyptus_sideroxylon:24.444437,eucalyptus_leucoxylon:24.444437,eucalyptus_camaldulensis:24.444437,eucalyptus_laeliae:24.444437,eucalyptus_delegatensis:24.444437,eucalyptus_regnans:24.444437,eucalyptus_globulus:24.444437,eucalyptus_ptychocarpa:24.444437,eucalyptus_robusta:24.444437,eucalyptus_gummifera:24.444437,eucalyptus_tereticornis:24.444437,eucalyptus_citriodora:24.444437)eucalyptus:24.444437,(eugenia_reinwardtiana:24.444437,eugenia_uniflora:24.444437,eugenia_brasiliensis:24.444437)eugenia:24.444437,feijoa_sellowiana:48.888874,(leptospermum_lanigerum:24.444437,leptospermum_grandiflorum:24.444437,leptospermum_scoparium:24.444437,leptospermum_sp.:24.444437,leptospermum_polygalifolium:24.444437)leptospermum:24.444437,(lophostemon_confertus:24.444437,lophostemon_grandiflorus:24.444437)lophostemon:24.444437,(melaleuca_quinquenervia:24.444437,melaleuca_squamea:24.444437,melaleuca_argentea:24.444437,melaleuca_armillaris:24.444437,melaleuca_leucadendra:24.444437,melaleuca_decora:24.444437,melaleuca_diosmatifolia:24.444437,melaleuca_viridiflora:24.444437,melaleuca_linariifolia:24.444437,melaleuca_bracteata:24.444437,melaleuca_thymifolia:24.444437,melaleuca_dealbata:24.444437)melaleuca:24.444437,myrcianthes_fragrans:48.888874,myrciaria_cauliflora:48.888874,pimenta_dioica:48.888874,(psidium_guajava:24.444437,psidium_littorale:24.444437)psidium:24.444437,(syzygium_jambos:24.444437,syzygium_luehmannii:24.444437,syzygium_tierneyanum:24.444437)syzygium:24.444437,taxandria_marginata:48.888874,tristaniopsis_laurina:48.888874,xanthostemon_chrysanthus:48.888874)myrtaceae:24.444437,(((ammania_auriculata:12.222219,ammania_coccinea:12.222219,ammania_robusta:12.222219,ammania_latifolia:12.222219)ammania:12.222219,(banksia_attenuata:12.222219,banksia_marginata:12.222219)banksia:12.222219,cuphea_lutea:24.444437,decodon_verticillatus:24.444437,lagerstroemia_indica:24.444437,(lythrum_lineare:12.222219,lythrum_salicaria:12.222219,lythrum_hyssopifolia:12.222219,lythrum_californicum:12.222219,lythrum_alatum:12.222219)lythrum:12.222219)lythraceae:12.222219,((epilobium_anustifolium:12.222219,epilobium_angustifolium:12.222219)epilobium:12.222219,(ludwigia_palustris:12.222219,ludwigia_hexapetala:12.222219)ludwigia:12.222219)onagraceae:12.222219)n00376:36.666656)n00377:21.666687,(((alliaria_petiolata:54.444439,berteroa_incana:54.444439,(brassica_napus:27.222219,brassica_oleracea:27.222219)brassica:27.222219,capsella_bursapastoris:54.444439,erysimum_cheiranthoides:54.444439,hesperis_matronalis:54.444439,sinapis_arvensis:54.444439)brassicaceae:27.222219,(brachychiton_populneus:40.833328,gossypium_hirsutum:40.833328)malvaceae:40.833328)n00341:5.333344,((((citrus_sinensis:16.500000,citrus_limon:16.500000,citrus_reticulata:16.500000)citrus:16.500000,(correa_sister_dawn:16.500000,correa_ivory_bells:16.500000,correa_backhouseana:16.500000,correa_alba:16.500000,correa_decumbens:16.500000,correa_reflexa:16.500000)correa:16.500000)rutaceae:16.500000,dodonaea_viscosa:49.500000)n00360:6.500000,mangifera_indica:56.000000)n00362:31.000000)n00363:8.000000)malvids:1.500000)eurosids:1.500000,vitis_vinifera:98.000000)rosids:20.000000,(myriophyllum_pinnatum:59.000000,myriophyllum_verrucosum:59.000000,myriophyllum_heterophyllum:59.000000,myriophyllum_spicatum:59.000000,myriophyllum_aquaticum:59.000000)myriophyllum:59.000000)n00495:7.000000,((((((((arctium_minus:26.201782,((carthamus_tinctorium:14.556549,(centaurea_maculosa:7.278275,centaurea_jacea:7.278275)centaurea:7.278275)n00125:8.733917,(cirsium_vulgare:18.632355,(cynara_cardunculus:9.316177,cynara_scolymus:9.316177)cynara:9.316177)n00130:4.658112)n00131:2.911316)n00139:2.911285,(onopordum_tauricum:14.556534,onopordum_acanthium:14.556534,onopordum_bractaetum:14.556534)onopordum:14.556534)n00143:11.852142,((cichorium_intybus:9.704346,hieracium_caespitosum:9.704346,lactuca_serriola:9.704346,sonchus_asper:9.704346,taraxacum_officinale:9.704346)cichorieae:25.605225,((helianthus_tuberosus:10.664673,jaumea_carnosa:10.664673)n00101:20.874451,(olearia_argophylla:11.038696,tanacetum_vulgare:11.038696)n00090:20.500427,(senecio_linearifolius:21.683167,tussilago_farfara:21.683167)n00089:9.855957)asteroideae:3.770447)n00110:5.655640)n00149:28.463379,stylidium_graminifolium:69.428589)n00162:25.988068,((billardiera_heterophylla:17.250000,bursaria_spinosa:17.250000,pittosporum_undulatum:17.250000)pittosporaceae:17.250000,((daucus_carota:11.500000,petroselinum_crispum:11.500000)apiaceae:11.500000,(polyscias_sambucifolia:11.500000,schefflera_actinophylla:11.500000)araliaceae:11.500000)n00082:11.500000)n00083:60.916656)n00168:9.250000,((((cordylanthus_maritimus:16.799988,(lavandula_dentata:8.399994,ocimum_basilicum:8.399994,origanum_vulgare:8.399994,prostanthera_lasianthos:8.399994)lamiaceae:8.399994)n00172:29.400024,(plantago_lanceolata:23.100006,plantago_maritima:23.100006)plantago:23.100006)n00184:38.744415,solanum_lycopersicum:84.944427)n00255:6.944458,((heliotropium_nicotianaefolium:30.629629,heliotropium_arborescens:30.629629,heliotropium_amplexicaule:30.629629)heliotropium:30.629629,myosotis_sylvatica:61.259258,symphytum_asperum:61.259258)boraginaceae:30.629629)n00259:12.777771)euasterids:6.166656,(rhododendron_colonel_coen:36.363647,vaccinium_sp.:36.363647)cassiopoideae_to_vaccinioideae:74.469666)n00281:8.166687,((((atriplex_prostrata:9.466084,atriplex_californica:9.466084)atriplex:9.466084,chenopodium_album:18.932169,sarcocornia_pacifica:18.932169)amaranthaceae:9.466084,spergularia_macrotheca:28.398254)n00300:55.601746,(frankenia_salina:46.666687,limonium_californicum:46.666687)n00310:37.333313)caryophyllales:35.000000)n00312:6.000000)n00496:16.000000,(adenanthos_obovatus:94.000000,grevillea_synapheae:94.000000,hakea_rostrata:94.000000,(isopogon_cuneatus:47.000000,isopogon_formosus:47.000000)isopogon:47.000000,lomatia_myricoides:94.000000,macadamia_tetraphylla:94.000000)proteaceae:47.000000)n00499:9.814819,ceratophyllum_demersum:150.814819)n00504:3.814819,(((((blyxa_octandra:30.060608,blyxa_aubertii:30.060608)blyxa:30.060608,egeria_densa:60.121216,elodea_canadensis:60.121216,hydrilla_verticillata:60.121216,hydrocharis_dubia:60.121216,najas_tenuifolia:60.121216,(ottelia_ovalifolia:30.060608,ottelia_alismoides:30.060608)ottelia:30.060608,(vallisneria_gigantea:30.060608,vallisneria_americana:30.060608,vallisneria_spiralis:30.060608)vallisneria:30.060608)hydrocharitaceae:30.060608,((potamogeton_crispus:28.181824,potamogeton_perfoliatus:28.181824,potamogeton_tricarinatus:28.181824)potamogeton:28.181824,triglochin_concinna:56.363647)n01373:33.818176)n01379:33.818176,((colocasia_esculenta:44.916656,pistia_stratiotes:44.916656)n01476:8.166656,peltandra_virginica:53.083313)n01478:70.916687)alismatales:11.222229,((((commelina_virginica:34.000000,murdannia_keisak:34.000000)commelinaceae:34.000000,(eichhornia_crassipes:34.000000,pontederia_cordata:34.000000)pontederiaceae:34.000000)commelinales:24.822205,(((distichlis_spicata:69.166656,(((microstegium_vimineum:38.936462,sorghum_bicolor:38.936462)n00884:5.562378,zea_mays:44.498840)n00929:16.687073,(panicum_hemitomon:30.592957,panicum_repens:30.592957)panicum:30.592957)n00952:7.980743)n00968:2.305573,(((lolium_arundinaceum:44.244690,(secale_cereale:30.630920,triticum_aestivum:30.630920)n01013:13.613770)n01019:3.403442,(poa_pratensis:42.353882,puccinellia_nutkaensis:42.353882)n01153:5.294250)n01155:21.176971,oryza_sativa:68.825104)n01317:2.647125)n01318:19.946808,sparganium_eurycarpus:91.419037)poales:1.403168)commelinids:19.822235,(lomandra_longifolia:26.750000,xanthorrhoea_preissii:26.750000)n01341:85.894440)n01352:22.577789)n01489:19.407410)n01490:3.814819,((atherosperma_moschatum:83.250000,(hedycarya_angustifolia:27.750000,((persea_americana:9.250000,persea_gratissima:9.250000)persea:9.250000,umbellularia_californica:18.500000)lauraceae:9.250000)n01491:55.500000)n01495:64.250000,tasmannia_lanceolata:147.500000)magnoliids:10.944458)n01504:10.944427,cabomba_caroliniana:169.388885)n01507:276.611115;
