# Supplementary material for: The Impact of Plant Enemies Shows a Phylogenetic Signal
Source: PLoS One. 2015 Apr 20;10(4):e0123758. doi: 10.1371/journal.pone.0123758 (PMC4404352; doi:10.1371/journal.pone.0123758)
Supplement: S1 Table — (PDF) [file pone.0123758.s003.pdf]

Table S1. Description of publications and study units from which data were used for phylogenetic analysis and meta-analysis.

Enemy	Type	Study Unit: Measured effect	Hosts	Publication
Bipolaris Mv isolate BP1	Fungal pathogen	4a: foliar lesion size	13	[1]
Bipolaris Mv isolate BP2	Fungal pathogen	4b: foliar lesion size	13	[1]
Bipolaris Mv isolate BP3	Fungal pathogen	4c: foliar lesion size	13	[1]
Bipolaris Mv isolate BP4	Fungal pathogen	4d: foliar lesion size	13	[1]
Colias philodice	Caterpillar	9a: pupal weight	6	[2]
Colias philodice	Caterpillar	9b: pupal development time	6	[2]
Trichoplusia ni	Caterpillar	12a: % larvae trenching, intact leaves	13	[3]
Trichoplusia ni	Caterpillar	12b: % larvae trenching, detached leaves	13	[3]
Spodoptera ornithogalli	Caterpillar	12c: larval mass gain, detached leaves	13	[3]
Spodoptera ornithogalli	Caterpillar	12d: larval mass gain, attached leaves	13	[3]
Trichoplusia ni	Caterpillar	12e: larval mass gain, detached leaves	13	[3]
Trichoplusia ni	Caterpillar	12f: larval mass gain, attached leaves	13	[3]
Schistocerca americana	Grasshopper	18a: area of leaf eaten	15	[4]
Procambarus acutus	Crayfish	22a: plant mass eaten	20	[5]
Procambarus spiculifer	Crayfish	22b: plant mass eaten	20	[5]

Larinus latus	Seed weevil	23b: % flower heads attacked	11	[6]
Tephritis postica	Seed fly	23c: % flower heads attacked	11	[6]
Terellia fuscicornis	Seed fly	23d: % flower heads attacked	11	[6]
Puccinia psidii MISOL	Fungal pathogen	24a: pustule density	8	[7]
Puccinia psidii PISOL	Fungal pathogen	24b: pustule density	8	[7]
Oxyops vitiosa	Foliar weevil	25a: leaf area eaten by larvae	25	[8]
Oxyops vitiosa	Foliar weevil	25b: leaf area eaten by adults	30	[8]
Oxyops vitiosa	Foliar weevil	25c: eggs laid	30	[8]
Galerucella pusilla	Leaf beetle	26a: eggs laid	13	[9]
Galerucella calmariensis	Leaf beetle	26b: eggs laid	13	[9]
Bagous hydrillae	Stem-boring weevil	27a: eggs laid	18	[10]
Cydia succedana	Pod moth	28a: eggs laid	10	[11]
Deuterocampta quadrijuga	Foliar beetle	31a: foliar damage rating	7	[12]
Dictyla sp.	Tingid bug	31c: foliar damage rating	7	[12]
Haplothrips heliotropica	Thrips	31d: foliar damage rating	7	[12]
Longitarsus sp.	Flea-beetle	31e: foliar damage rating	7	[12]
Phytophthora ramorum	Oomycete pathogen	32a: disease severity, winter no wound	41	[13]

Phytophthora ramorum	Oomycete pathogen	32b: disease severity, summer no wound	64	[13]
Phytophthora ramorum	Oomycete pathogen	32c: disease severity, summer wound	64	[13]
Phytophthora ramorum	Oomycete pathogen	32d: leaf infection, winter no wound	42	[13]
Phytophthora ramorum	Oomycete pathogen	32e: leaf infection, summer no wound	65	[13]
Phytophthora ramorum	Oomycete pathogen	32f: leaf infection, summer wound	65	[13]
Phytophthora ramorum	Oomycete pathogen	32g: susceptibility rating	65	[13]
Phytophthora ramorum	Oomycete pathogen	32h: sporangium production	28	[13]
Phytophthora ramorum	Oomycete pathogen	32i: lesion area	28	[13]
Phytophthora ramorum	Oomycete pathogen	34a: lesion length	63	[14]
Drosophila suzukii	Fruit fly	35a: adult female mass	7	[15]
Drosophila suzukii	Fruit fly	35b: fly development time	7	[15]
Drosophila suzukii	Fruit fly	35c: flight bioassay	7	[15]
Drosophila suzukii	Fruit fly	35d: oviposition per fruit	7	[15]
Drosophila suzukii	Fruit fly	35e: percent fly emergence	7	[15]
Drosophila suzukii	Fruit fly	35f: oviposition frequency	7	[15]
Drosophila suzukii	Fruit fly	35g: Host Potential Index	13	[15]

1. Kleczewski NM, Flory SL, Clay K. Variation in pathogenicity and host range of *Bipolaris* sp. causing leaf blight disease on the invasive grass *Microstegium vimineum*. Weed Sci. 2012;60(3):486-93.
2. Tabashnik BE. Host range evolution - the shift from native legume hosts to alfalfa by the butterfly, *Colias philodice-eriphyle*. Evolution. 1983;37(1):150-62.
3. Dussourd DE, Denno RF. Host-range of generalist caterpillars - trenching permits feeding on plants with secretory canals. Ecology. 1994;75(1):69-78.
4. Jogesh T, Carpenter D, Cappuccino N. Herbivory on invasive exotic plants and their non-invasive relatives. Biol Invasions. 2008;10(6):797-804.
5. Parker JD, Hay ME. Biotic resistance to plant invasions? Native herbivores prefer non-native plants. Ecol Lett. 2005;8(9):959-67.
6. Briese DT, Sheppard AW, Reifenberg JM. Open-field host-specificity testing for potential biological-control agents of *Onopordum* thistles. Biol Control. 1995;5(2):158-66.
7. Rayachhetry MB, Van TK, Center TD, Elliott ML. Host range of *Puccinia psidii*, a potential biological control agent of *Melaleuca quinquenervia* in Florida. Biol Control. 2001;22(1):38-45.
8. Balciunas JK, Burrows DW, Purcell MF. Field and laboratory host ranges of the Australian weevil, *Ocyops vitiosa* (Coleoptera, Curculionidae), a potential biological-control agent for the paperbark tree, *Melaleuca quinquenervia*. Biol Control. 1994;4(4):351-60.
9. Blossey B, Schroeder D, Hight SD, Malecki RA. Host-specificity and environmental-impact of 2 leaf-beetles (*Galerucella calmariensis* and *G. pusilla*) for biological control of purple-loosestrife (*Lythrum salicaria*). Weed Sci. 1994;42(1):134-40.
10. Balciunas JK, Burrows DW. Distribution, abundance and field host-range of *Hydrellia balciunasi* Bock (Diptera: Ephydridae) a biological control agent for the aquatic weed *Hydrilla verticillata* (Lf) Royle. Aust J Entomol. 1996;35:125-30.
11. Withers T, Hill R, Paynter Q, Fowler S, Gourlay H. Post-release investigations into the field host range of the gorse pod moth *Cydia succedana* Denis & Schiffermüller (Lepidoptera: Tortricidae) in New Zealand. N Z Entomol. 2008;31(1):67-76.
12. Briese DT, Zapater M, Andorno A, Perez-Camargo G. A two-phase open-field test to evaluate the host-specificity of candidate biological control agents for *Heliotropium amplexicaule*. Biol Control. 2002;25(3):259-72.
13. Ireland KB, Huberli D, Dell B, Smith IW, Rizzo DM, Hardy G. Potential susceptibility of Australian flora to a NA2 isolate of *Phytophthora ramorum* and pathogen sporulation potential. For Pathol. 2012;42(4):305-20.
14. Ireland KB, Huberli D, Dell B, Smith IW, Rizzo DM, Hardy G. Potential susceptibility of Australian native plant species to branch dieback and bole canker diseases caused by *Phytophthora ramorum*. Plant Pathol. 2012;61(2):234-46.
15. Bellamy DE, Sisterson MS, Walse SS. Quantifying host potentials: Indexing postharvest fresh fruits for spotted wing *Drosophila*, *Drosophila suzukii*. Plos One. 2013;8(4).
