# Supplementary material for: The Impact of Plant Enemies Shows a Phylogenetic Signal
Source: PLoS One. 2015 Apr 20;10(4):e0123758. doi: 10.1371/journal.pone.0123758 (PMC4404352; doi:10.1371/journal.pone.0123758)
Supplement: S2 Table — Regressions take the form of RI = b •0 + b •1log10(PD + 1). Phylogenetic distance (PD) is the estimated time of independent evolution from the most affected species in the study unit (in Ma, millions of years); b0 is the slope and b1 is the intercept. P-values in bold indicate slopes significantly different from zero (alpha = 0.05). Confidence intervals (95%) are given for the slope of each model. The type of interaction and which partner was impacted are used for meta-analysis grouping in Table 1 and Fig 1 in the main text. (PDF) [file pone.0123758.s004.pdf]

Table S2. Regression outputs from linear regression for each of the study units described in Table S1. Regressions take the form of $RI = b_{\cdot 0} + b_{\cdot 1}\log_{10}(PD + 1)$. Phylogenetic distance (PD) is the estimated time of independent evolution from the most affected species in the study unit (in Ma, millions of years); b_0 is the slope and b_1 is the intercept. P-values in bold indicate slopes significantly different from zero (alpha = 0.05). Confidence intervals (95%) are given for the slope of each model. The type of interaction and which partner was impacted are used for meta-analysis grouping in Table 1 and Figure 1 in the main text.

Unit	Intercept	SE int	Slope	SE slope	*t*-value	P-value	df	adjR^2	CI025	CI975	Interaction	Impacted
**24a**	1.0000	0.1835	-0.3748	0.0984	-3.8108	**0.00885**	6	0.6589	-0.6155	-0.1341	disease	plant
**24b**	1.0119	0.2132	-0.3561	0.1166	-3.0550	**0.02236**	6	0.5435	-0.6414	-0.0709	disease	plant
**32a**	0.9945	0.0744	-0.3817	0.0307	-12.4214	**3.93E-15**	39	0.7931	-0.4439	-0.3196	disease	plant
**32b**	1.0573	0.1793	-0.3758	0.0742	-5.0656	**3.92E-06**	62	0.2813	-0.5241	-0.2275	disease	plant
**32c**	0.9359	0.1723	-0.3322	0.0777	-4.2761	**0.00006**	62	0.2153	-0.4875	-0.1769	disease	plant
**32d**	0.7726	0.2795	-0.0095	0.1195	-0.0795	0.93699	40	-0.0248	-0.2510	0.2320	disease	plant
**32e**	1.2158	0.2809	-0.2016	0.1214	-1.6615	0.10157	63	0.0268	-0.4442	0.0409	disease	plant
**32f**	1.0412	0.2022	-0.0651	0.0875	-0.7440	0.45965	63	-0.0070	-0.2400	0.1098	disease	plant
**32g**	0.8616	0.1616	-0.2162	0.0744	-2.9068	**0.00503**	63	0.1043	-0.3649	-0.0676	disease	plant
**32i**	0.9909	0.0596	-0.3997	0.0252	-15.8305	**3.93E-15**	26	0.9024	-0.4516	-0.3478	disease	plant
**34a**	0.9620	0.1127	-0.3637	0.0472	-7.7020	**3.92E-06**	61	0.4847	-0.4581	-0.2693	disease	plant

**4a**	1.2513	0.3031	-0.4508	0.1373	-3.2824	**0.00730**	11	0.4489	-0.7531	-0.1485	disease	plant
**4b**	1.1304	0.3180	-0.4117	0.1441	-2.8575	**0.01558**	11	0.3739	-0.7289	-0.0946	disease	plant
**4c**	0.9595	0.0415	-0.3955	0.0188	-21.0900	**3.93E-15**	11	0.9737	-0.4368	-0.3542	disease	plant
**4d**	0.9595	0.0415	-0.3955	0.0188	-21.0900	**3.92E-06**	11	0.9737	-0.4368	-0.3542	disease	plant
**18a**	0.9271	0.2392	-0.1648	0.1087	-1.5160	0.15344	13	0.0849	-0.3996	0.0700	herbivory	plant
**22a**	0.8737	0.3156	-0.1704	0.1361	-1.2516	0.22674	18	0.0290	-0.4564	0.1156	herbivory	plant
**22b**	0.8396	0.1732	-0.1809	0.0758	-2.3871	**0.02816**	18	0.1983	-0.3402	-0.0217	herbivory	plant
**23b**	1.0383	0.1732	-0.4981	0.1162	-4.2881	**0.00202**	9	0.6349	-0.7609	-0.2353	herbivory	plant
**23c**	0.5241	0.2332	-0.2683	0.1564	-1.7160	0.12030	9	0.1628	-0.6221	0.0854	herbivory	plant
**23d**	1.1577	0.2152	-0.5863	0.1427	-4.1086	**0.00264**	9	0.6136	-0.9091	-0.2635	herbivory	plant
**25a**	0.8917	0.1268	-0.4078	0.0642	-6.3549	**1.75E-06**	23	0.6214	-0.5405	-0.2750	herbivory	plant
**25b**	0.9509	0.2452	-0.3867	0.1231	-3.1422	**0.00393**	28	0.2343	-0.6388	-0.1346	herbivory	plant
**31a**	1.0673	0.3282	-0.4497	0.1721	-2.6135	**0.04746**	5	0.4928	-0.8920	-0.0074	herbivory	plant
**31c**	1.0415	0.2490	-0.4516	0.1306	-3.4592	**0.01805**	5	0.6464	-0.7872	-0.1160	herbivory	plant
**31d**	0.9957	0.1194	-0.4551	0.0626	-7.2710	**0.00076**	5	0.8963	-0.6159	-0.2942	herbivory	plant
**31e**	1.0783	0.2123	-0.4489	0.1113	-4.0324	**0.00999**	5	0.7178	-0.7350	-0.1627	herbivory	plant
**32h**	0.9965	0.1140	-0.4042	0.0483	-8.3759	**7.39E-09**	26	0.7192	-0.5034	-0.3050	disease	enemy development

**12c**	0.9728	0.1567	-0.1663	0.0689	-2.4136	**0.03439**	11	0.2868	-0.3179	-0.0146	herbivory	enemy development
**12d**	0.7731	0.3524	-0.1135	0.1611	-0.7046	0.49573	11	-0.0438	-0.4680	0.2410	herbivory	enemy development
**12e**	0.9983	0.1783	-0.2369	0.0784	-3.0205	**0.01164**	11	0.4037	-0.4095	-0.0643	herbivory	enemy development
**12f**	0.9990	0.1754	-0.2655	0.0771	-3.4413	**0.00551**	11	0.4747	-0.4353	-0.0957	herbivory	enemy development
**35a**	0.9983	0.0697	-0.0781	0.0361	-2.1637	0.08279	5	0.3803	-0.1710	0.0147	herbivory	enemy development
**35b**	0.9983	0.0368	-0.0629	0.0172	-3.6542	**0.01468**	5	0.6731	-0.1071	-0.0187	herbivory	enemy development
**35e**	1.0514	0.3257	-0.2953	0.1653	-1.7864	0.13408	5	0.2675	-0.7201	0.1296	herbivory	enemy development
**9a**	0.9888	0.0542	-0.0995	0.0495	-2.0110	0.11467	4	0.3784	-0.2369	0.0379	herbivory	enemy development
**9b**	0.9016	0.0718	-0.0300	0.0656	-0.4566	0.67167	4	-0.1881	-0.2121	0.1522	herbivory	enemy development
**12a**	1.2038	0.3521	-0.3916	0.1685	-2.3238	**0.04030**	11	0.2683	-0.7624	-0.0207	herbivory	enemy behavior
**12b**	1.0660	0.2286	-0.4119	0.1094	-3.7655	**0.00312**	11	0.5234	-0.6527	-0.1712	herbivory	enemy behavior
**25c**	0.8061	0.1127	-0.3768	0.0566	-6.6602	**3.15E-07**	28	0.5992	-0.4926	-0.2609	herbivory	enemy behavior
**26a**	0.7867	0.1166	-0.4439	0.0709	-6.2567	**0.00004**	12	0.7458	-0.5985	-0.2893	herbivory	enemy behavior
**26b**	0.8379	0.1148	-0.4817	0.0710	-6.7813	**0.00003**	11	0.7894	-0.6381	-0.3254	herbivory	enemy behavior
**27a**	1.1011	0.1785	-0.4031	0.0829	-4.8620	**0.00017**	16	0.5711	-0.5788	-0.2273	herbivory	enemy behavior
**28a**	1.0459	0.1185	-0.4596	0.0838	-5.4824	**0.01193**	3	0.8790	-0.7264	-0.1928	herbivory	enemy behavior
**35c**	1.0231	0.0603	-0.0879	0.0312	-2.8125	**0.03743**	5	0.5353	-0.1682	-0.0076	herbivory	enemy behavior

**35d**	0.9973	0.0974	-0.4063	0.0494	-8.2173	**0.00043**	5	0.9173	-0.5335	-0.2792	herbivory	enemy behavior
**35f**	0.9560	0.3470	-0.2026	0.1761	-1.1507	0.30189	5	0.0512	-0.6554	0.2501	herbivory	enemy behavior
**35g**	1.0926	0.2173	-0.1799	0.1085	-1.6589	0.12535	11	0.1274	-0.4187	0.0588	herbivory	enemy behavior
